# Association between abdominal CT-based body composition parameters and early diabetic kidney disease in type 2 diabetes mellitus: a retrospective cross-sectional study

**DOI:** 10.7717/peerj.20535

**Published:** 2026-01-15

**Authors:** Yinying Tang, Xinyi Cen, Qi Dai, Hai Chen, Jie Zhang, Fangfang Zhou, Jianjun Zheng, Jingfeng Zhang

**Affiliations:** 1 Department of Radiology, Ningbo No. 2 Hospital, Ningbo, Zhejiang, China; 2 Health Science Center, Ningbo University, Ningbo, Zhejiang, China; 3 Department of Endocrinology, Ningbo No. 2 Hospital, Ningbo, Zhejiang, China; 4 Department of Nephrology, Ningbo No. 2 Hospital, Ningbo, Zhejiang, China

**Keywords:** Type 2 diabetes mellitus, Diabetic kidney disease, Body composition analysis, Computed tomography, Renal sinus fat

## Abstract

**Background:**

Early identification of diabetic kidney disease (DKD) in type 2 diabetes mellitus (T2DM) remains challenging due to limitations of conventional biomarkers. Body composition analysis using computed tomography (CT) may provide novel insights into DKD risk stratification.

**Objective:**

To investigate the association between abdominal CT-based body composition parameters and early diagnosis of DKD in patients with T2DM.

**Methods:**

This retrospective cohort study enrolled 310 patients with T2DM from the Second Hospital of Ningbo between January 2020 and December 2024. Patients were stratified into the early DKD group (*n* = 131) and the T2DM control group without DKD (*n* = 179) based on the results of renal function assessment. Using Slice-O-Matic software, we measured area, index, and radiodensity of skeletal muscle and adipose tissue depots at the L3 vertebral level on abdominal CT images. Spearman correlation analysis evaluated associations between body composition parameters and renal function indicators. Univariate and multivariate logistic regression analyses identified independent risk factors for the development of early DKD. Receiver operating characteristic (ROC) curve analysis was employed to assess the predictive value of body composition parameters for early DKD.

**Results:**

Multivariate logistic regression analysis revealed four independent risk factors of early DKD. Age (OR = 1.03, 95% CI [1.01–1.06], *P* = 0.044), high-sensitivity C-reactive protein (OR = 1.02, 95% CI [1.01–1.04], *P* = 0.005), renal sinus fat index (OR = 0.50, 95% CI [0.30–0.85], *P* = 0.010), and renal sinus fat density (OR = 0.79, 95% CI [0.74–0.85], *P* < 0.001). Multiple linear regression analysis demonstrated that renal sinus fat density maintained significant associations with both the urinary albumin-to-creatinine ratio (β = −1.88, *P* < 0.001) and the estimated glomerular filtration rate (β = 0.22, *P* = 0.017) after adjusting for confounding variables. The combined clinical-body composition model (AUC = 0.81, 95% CI [0.76–0.86]) and the body composition-only model (AUC = 0.77, 95% CI [0.72–0.82]) both demonstrated superior predictive performance compared to the clinical-only model (AUC = 0.67, 95% CI [0.61–0.73]).

**Conclusions:**

Reduced renal sinus fat density is significantly associated with early DKD in T2DM patients, demonstrating potential utility as an imaging biomarker for risk stratification. These findings support the integration of CT-based body composition analysis into comprehensive DKD screening strategies.

## Introduction

Diabetic kidney disease (DKD) represents the most devastating microvascular complication of type 2 diabetes mellitus (T2DM) and constitutes the leading cause of end-stage renal disease globally (Meir et al., 2024). Epidemiological evidence indicates that approximately 40% of T2DM patients eventually progress to DKD, with a substantial proportion advancing to end-stage renal disease, significantly compromising patient outcomes and quality of life (Umanath & Lewis, 2018). The clinical and economic burden of DKD continues to escalate worldwide, necessitating improved strategies for early detection and intervention.

Current diagnostic paradigms for DKD primarily rely on microalbuminuria detection and estimated glomerular filtration rate (eGFR) assessment (Ning et al., 2020). However, these conventional biomarkers exhibit inherent limitations in sensitivity, often failing to accurately identify early pathological changes before irreversible renal damage occurs (Thomas et al., 2009). Notably, emerging evidence suggests that selected patients with early DKD may achieve disease stabilization or even reversal through timely therapeutic interventions (Beyerstedt et al., 2025), underscoring the critical importance of establishing more sensitive and precise early diagnostic frameworks.

Obesity has been established as a pivotal risk factor for T2DM development and progression (Chandrasekaran & Weiskirchen, 2024). Compared to generalized obesity, abnormal abdominal fat distribution patterns demonstrate stronger associations with DKD occurrence in T2DM patients (Hu et al., 2016). Computed tomography (CT) technology enables precise quantification of regional fat distribution based on tissue radiodensity differences. It is widely recognized as the imaging gold standard for body composition assessment (Tolonen et al., 2021). Beyond accurate measurement of adipose tissue area and volume at various anatomical sites, CT facilitates indirect evaluation of adipose tissue quality characteristics through density analysis.

Furthermore, sarcopenia has emerged as an essential risk factor for insulin resistance, with growing recognition of its association with diabetic complications (Son et al., 2017). Recent investigations demonstrate that CT-measured adipose tissue density can indirectly reflect metabolic activity and inflammatory status of fat depots. For instance, pericoronary adipose tissue density has been validated as a non-invasive marker for coronary inflammation and cardiovascular risk assessment (Zhihong et al., 2025).

Building on this scientific foundation, the present study aimed to systematically evaluate body composition characteristics in T2DM patients using abdominal CT imaging technology. We comprehensively assessed muscle tissue and regional adipose tissue parameters, including area, indices, and radiodensity (measured in Hounsfield Units (HU), a quantitative measure of tissue density that reflects tissue quality and composition), to investigate their associations with early DKD development risk, thereby providing novel imaging-based evidence for early DKD identification and risk stratification.

## Materials and Methods

### Study design and participants

This retrospective cross-sectional study analyzed baseline data from T2DM patients who received inpatient care and underwent abdominal CT examination at the Second Hospital of Ningbo between January 2020 and December 2024.

Inclusion criteria: (1) T2DM diagnosis according to Chinese Diabetes Society criteria (Chinese Diabetes Society, 2025); (2) Normal renal function (urinary albumin-to-creatinine ratio (UACR) < 30 mg/g with eGFR ≥ 60 mL/min/1.73 m^2^) sustained for ≥3 months, or mild renal dysfunction (30 ≤ UACR < 300 mg/g with eGFR ≥ 60 ml/min/1.73 m^2^, or UACR < 30 mg/g with 45 ≤ eGFR < 60 ml/min/1.73 m^2^) sustained for ≥3 months (Kidney Disease: Improving Global Outcomes (KDIGO) Diabetes Work Group, 2022). Exclusion criteria: (1) Incomplete clinic data or inadequate CT image quality; (2) Patients with hyperthyroidism or hypothyroidism; (3) Patients with a history of malignant tumors; (4) Patients with a history of congenital isolated or polycystic kidney disease; (5) Patients with a history of other kidney diseases.

**Figure 1:**
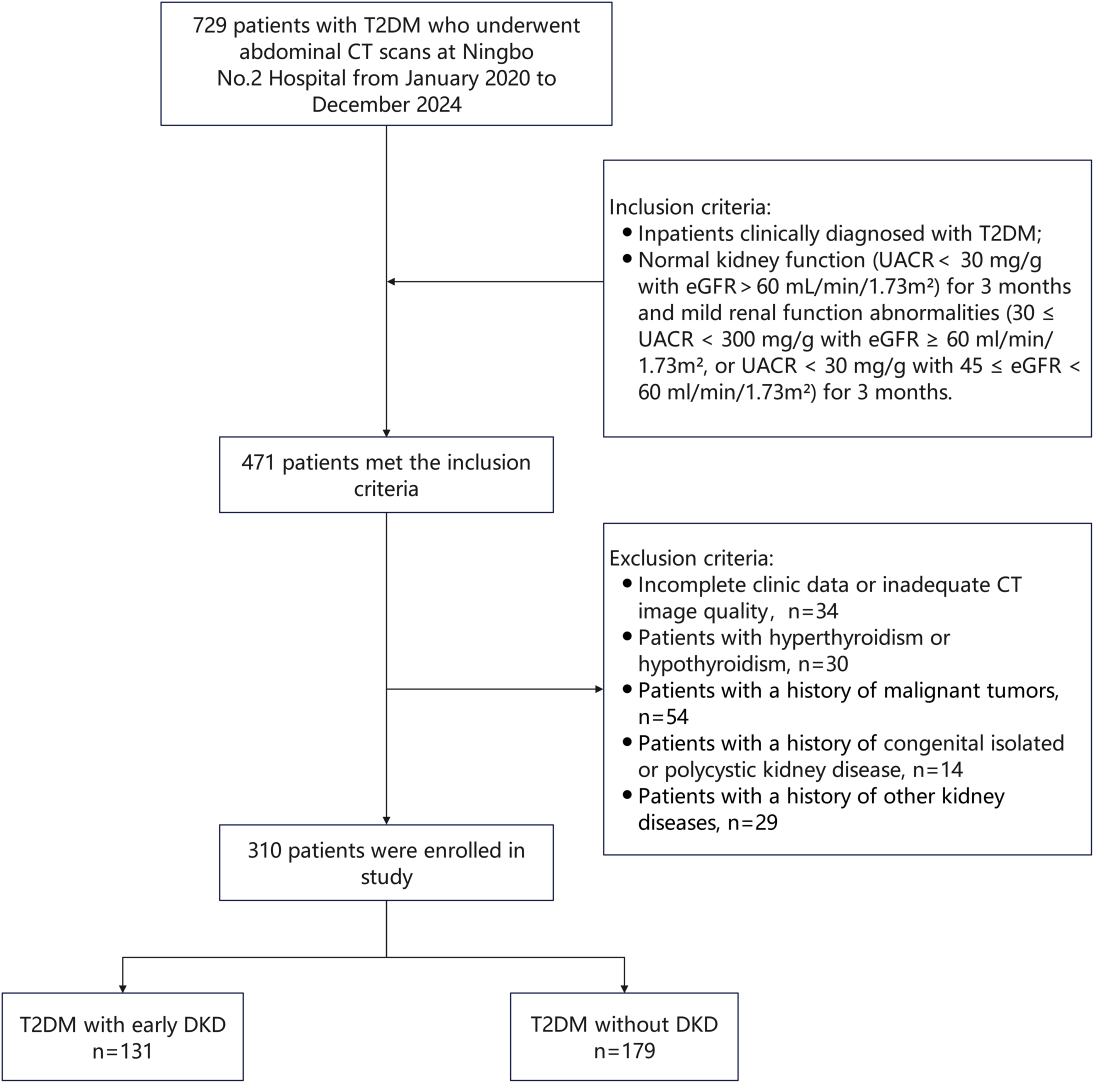
The patient selection flowchart. CT, computed tomography; DKD, diabetic kidney disease; T2DM, type 2 diabetes mellitus.

Based on these criteria, 310 eligible T2DM patients were enrolled and stratified into two groups according to renal function status: T2DM without DKD group (normal renal function: UACR < 30 mg/g with eGFR ≥ 60 mL/min/1.73 m^2^, *n* = 179) and T2DM with early DKD group (mild renal dysfunction: 30 ≤ UACR < 300 mg/g with eGFR ≥ 60 ml/min/1.73 m^2^, or UACR < 30 mg/g with 45 ≤ eGFR < 60 ml/min/1.73 m^2^, *n* = 131). Figure 1 shows the patient selection flowchart.

The study protocol received approval from the Ethics Committee of the Second Hospital of Ningbo (Ethics approval number: PJ-NBEY-KY-2025-014-01). Informed consent was waived due to the retrospective nature of the study.

### Clinical data collection

Demographic characteristics and clinical data of patients were collected through the hospital’s electronic medical record system, encompassing baseline characteristics (sex, age, height, weight, body mass index (BMI), history of hypertension, smoking status, alcohol use, and current medications including SGLT2 inhibitors, metformin, alpha-glucosidase inhibitors, insulin secretagogue, thiazolidinediones, insulin, and DPP-4 Inhibitors) and laboratory parameters, including renal function (estimated glomerular filtration rate (eGFR), urea, uric acid, and urinary albumin-to-creatinine ratio (UACR)), lipid profile (total cholesterol (TC), triglycerides (TG), low-density lipoprotein cholesterol (LDL-C), and high-density lipoprotein cholesterol (HDL-C)), inflammatory markers (high-sensitivity C-reactive protein (CRP)), and glycemic control indicators (glycated hemoglobin (HbA1c) and serum C-peptide).

BMI was calculated as weight (kg) divided by height squared (m^2^). eGFR was determined using the Chronic Kidney Disease Epidemiology Collaboration (CKD-EPI) equation.

### CT imaging protocol

Abdominal CT examinations were performed using a 96-slice dual-source dual-detector CT scanner (SOMATOM Force, Siemens Healthineers, Erlangen, Germany). Standardized scanning parameters included: tube voltage 110 kV, automatic tube current modulation, pitch 1.2 mm, slice spacing 5 mm, slice thickness 5 mm, matrix 512 × 512, scan field of view (SFOV) 50 cm, display field of view (DFOV) 39 cm, and gantry rotation time 2.88 s. Patients were positioned supine and examined during end-inspiratory breath-hold for unenhanced abdominal CT acquisition.

### Body composition analysis

A trained radiologist, blinded to clinical information, used Slice-O-Matic software (version 5.0, TomoVision, Montreal, Canada) to measure skeletal muscle (SM), intermuscular adipose tissue (IMAT), visceral adipose tissue (VAT) and subcutaneous adipose tissue (SAT) at the mid-L3 vertebral level, and to measure perirenal adipose tissue (PAT) and renal sinus fat (RSF) at the level of the renal artery entering the renal sinus (Cheng et al., 2018; Wagner et al., 2012). The measurements were performed using semi-automatic segmentation based on CT density thresholds. For both PAT and RSF, the left and right kidneys were contoured separately, and the average values were calculated for analysis.

Measured parameters included: (1) SM: Psoas, erector spinae, quadratus lumborum, transverse abdominis, external oblique, internal oblique, and rectus abdominis muscles; (2) IMAT: Fat within abdominal wall skeletal muscle interfaces; (3) VAT: Intra-abdominal fat surrounding organs; (4) SAT: Fat between skin and abdominal wall muscles; (5) PAT: Fat between anterior and posterior renal fascia; (6) RSF: Adipose tissue within the renal sinus. Tissue density thresholds: SM: −29 to 150 HU; VAT, PAT, RSF: −150 to −50 HU; SAT, IMAT: −190 to −30 HU (Fan et al., 2024; Chen et al., 2021).

Body composition indices were calculated by dividing cross-sectional tissue areas by height squared, yielding visceral adipose tissue index (VATI), subcutaneous adipose tissue index (SATI), intermuscular adipose tissue index (IMATI), skeletal muscle index (SMI), perirenal adipose tissue index (PATI), and renal sinus fat index (RSFI). Mean tissue radiodensity values were simultaneously recorded. The mean radiodensities were collected from the same regions of interest used for body composition areas (subcutaneous adipose tissue density (SATd), visceral adipose tissue density (VATd), intermuscular adipose tissue (IMATd), skeletal muscle area density (SMAd), perirenal adipose tissue density (PATd), and renal sinus fat density (RSFd)).

**Figure 2:**
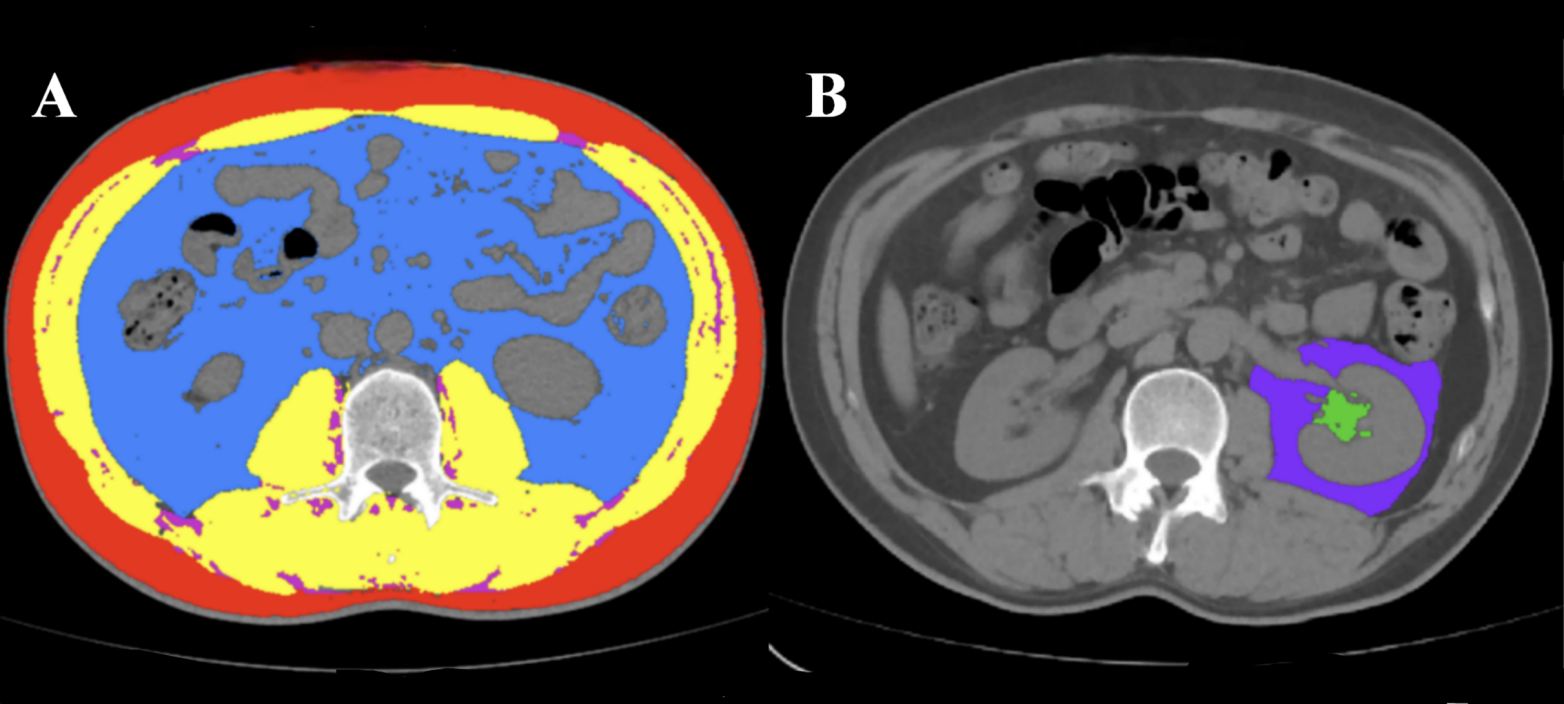
Representative cross-sectional CT images and muscle and fat of each part. (A) SAT (red), VAT (blue), SM (yellow) and IIMAT (magenta); (B) RSF (green) and PAT (purple).

To assess measurement reliability, an independent senior radiologist performed duplicate measurements on randomly selected images from 30 patients for inter-observer agreement evaluation. The body composition drawing is shown in Fig. 2.

### Statistical analysis

Statistical analyses were performed using Zstats software (www.zstats.net) and R software (version 4.3.3). The normality of data distribution was assessed using the Kolmogorov-Smirnov test. For descriptive statistics, normally distributed continuous variables were presented as mean ± standard deviation ( ${{\bar{\rm  x}}\pm s}$), non-normally distributed variables as median (interquartile range) [M(Q1, Q3)], and categorical variables as frequencies (percentages). Mean imputation was applied to continuous variables exhibiting less than 5% missing data. In terms of between-group comparisons, the independent samples t-test was used for normally distributed continuous variables, the Mann-Whitney U test for non-normally distributed variables, and the $\chi^2$ test or Fisher’s exact test for categorical variables. Correlation analysis was conducted using Spearman correlation to evaluate the associations between body composition parameters and renal function indicators (eGFR, UACR). The criteria for correlation strength were as follows: |r| ≥ 0.9 indicated a strong correlation, 0.7 ≤ |r| < 0.9 a moderate-to-strong correlation, 0.4 ≤ |r| < 0.7 a moderate correlation, 0.2 ≤ |r| < 0.4 a weak correlation, and |r| < 0.2 a negligible correlation. For regression analysis, variables with a *p*-value of less than 0.05 in the univariate analysis were included in the multivariate logistic regression using stepwise selection to identify independent risk factors for early DKD. Considering potential influences of gender, age (<45, ≥45), and hypertension status on body composition, stratified analyses were performed. The performance of the predictive model was evaluated by constructing receiver operating characteristic (ROC) curves and calculating the area under the curve (AUC). Statistical significance of the difference between AUC using various indicators was calculated by DeLong’s test (Table S1). Intraclass correlation coefficients (ICCs) were used to assess the reproducibility of inter-observer measurements. The interpretation of ICC values was as follows: <0.50 indicated poor agreement, 0.50–0.75 moderate agreement, 0.75–0.90 good agreement, and >0.90 excellent agreement. All statistical tests were two-sided, with a *P*-value of less than 0.05 considered statistically significant.

## Results

### Baseline characteristics

The study cohort comprised 310 T2DM patients: 189 males (61.0%) and 121 females (39.0%), with a mean age of 54 years. According to renal function status, the study included two groups: early DKD (*n* = 131) and non-DKD (*n* = 179). Baseline comparisons revealed that early DKD patients were significantly older (58.00 *vs* 52.00 years, *P* < 0.001) with higher hypertension prevalence (64.12% *vs* 39.11%, *P* < 0.001). Body composition analysis revealed that patients with early DKD had significantly higher VATII (56.02 *vs* 51.76, *P* = 0.049), IMAT (3.42 *vs* 3.00, *P* = 0.023), RSFI (1.21 *vs* 0.85, *P* < 0.001), and PATI (16.37 *vs* 13.52, *P* = 0.002) compared to the non-DKD group. Other clinical biochemical parameters and remaining body composition variables showed no significant between-group differences (*P* > 0.05). Detailed baseline characteristics are presented in Table 1.

**Table 1:** Baseline characteristics of the population.

Variables	Total (*n* = 310)	T2DM without DKD (*n* = 179)	T2DM with early DKD (*n* = 131)	Statistic	*P*
Age, M (Q_1_, Q_3_)	54.000 (46.000, 63.000)	52.000 (45.000, 61.000)	58.000 (47.500, 66.500)	Z = −3.428	<0.001
Height, M (Q_1_, Q_3_)	1.660 (1.600, 1.720)	1.680 (1.600, 1.730)	1.650 (1.595, 1.720)	Z = −0.978	0.328
Weight, M (Q_1_, Q_3_)	69.500 (60.000, 80.000)	67.000 (60.000, 76.000)	70.000 (60.500, 80.000)	Z = −0.953	0.341
BMI, M (Q_1_, Q_3_)	24.660 (22.205, 27.585)	24.340 (21.880, 27.115)	24.890 (22.805, 28.230)	Z = −1.530	0.126
**Hypertension, *n* (%)**				χ² = 18.935	<0.001
No	156 (50.323)	109 (60.894)	47 (35.878)		
Yes	154 (49.677)	70 (39.106)	84 (64.122)		
**Smoking, *n* (%)**				χ² = 0.124	0.725
No	228 (73.548)	133 (74.302)	95 (72.519)		
Yes	82 (26.452)	46 (25.698)	36 (27.481)		
**Alcohol consumption, *n* (%)**				χ² = 0.689	0.406
No	255 (82.258)	150 (83.799)	105 (80.153)		
Yes	55 (17.742)	29 (16.201)	26 (19.847)		
**Gender, *n* (%)**				χ² = 0.001	0.975
Female	121 (39.032)	70 (39.106)	51 (38.931)		
Male	189 (60.968)	109 (60.894)	80 (61.069)		
**Pre-admission medication**					
SGLT2 inhibitors, *n* (%)				χ² = 2.368	0.124
No	227 (73.226)	137 (76.536)	90 (68.702)		
Yes	83 (26.774)	42 (23.464)	41 (31.298)		
Metformin, *n* (%)				χ² = 3.182	0.074
No	186 (60.000)	115 (64.246)	71 (54.198)		
Yes	124 (40.000)	64 (35.754)	60 (45.802)		
Alpha-glucosidase inhibitors, *n* (%)				χ² = 1.395	0.238
No	262 (84.516)	155 (86.592)	107 (81.679)		
Yes	48 (15.484)	24 (13.408)	24 (18.321)		
Insulin secretagogue, *n* (%)				χ² = 1.461	0.227
No	177 (57.097)	97 (54.190)	80 (61.069)		
Yes	133 (42.903)	82 (45.810)	51 (38.931)		
Thiazolidinediones, *n* (%)				χ² = 0.032	0.858
No	300 (96.774)	174 (97.207)	126 (96.183)		
Yes	10 (3.226)	5 (2.793)	5 (3.817)		
Insulin, *n* (%)				χ² = 0.255	0.614
No	264 (85.161)	154 (86.034)	110 (83.969)		
Yes	46 (14.839)	25 (13.966)	21 (16.031)		
DPP-4 inhibitors, *n* (%)				$\chi^2$ = 1.666	0.197
No	260 (83.871)	146 (81.564)	114 (87.023)		
Yes	50 (16.129)	33 (18.436)	17 (12.977)		
Systolic pressure, Mean ± SD	133.422 ± 16.920	132.207 ± 16.053	135.082 ± 17.967	t = −1.481	0.140
Diastolic pressure, Mean ± SD	79.705 ± 11.374	79.497 ± 10.893	79.990 ± 12.037	t = −0.376	0.707
eGFR, Mean ± SD	90.006 ± 11.648	90.833 ± 11.141	88.875 ± 12.260	t = 1.465	0.144
TG, M (Q_1_, Q_3_)	1.550 (1.028, 2.060)	1.510 (0.940, 2.085)	1.710 (1.145, 2.025)	Z = −1.506	0.132
TC, M (Q_1_, Q_3_)	4.687 (3.925, 5.310)	4.740 (4.005, 5.380)	4.687 (3.895, 5.025)	Z = −1.388	0.165
LDL-C, M (Q_1_, Q_3_)	2.821 (2.245, 3.328)	2.870 (2.300, 3.420)	2.821 (2.240, 3.085)	Z = −1.634	0.102
HDL-C, M (Q_1_, Q_3_)	1.130 (0.940, 1.290)	1.120 (0.975, 1.295)	1.147 (0.915, 1.280)	Z = −1.146	0.252
Urea, M (Q_1_, Q_3_)	5.550 (4.380, 6.680)	5.520 (4.560, 6.490)	5.630 (4.215, 7.110)	Z = −0.321	0.748
Uric acid, M (Q_1_, Q_3_)	293.750 (238.700, 357.975)	289.000 (238.450, 349.700)	297.000 (239.050, 370.950)	Z = −0.736	0.462
Serum C-Peptide, M (Q_1_, Q_3_)	1.720 (0.975, 2.647)	1.670 (1.000, 2.495)	1.790 (0.935, 2.833)	Z = −0.332	0.740
HbA1c, M (Q_1_, Q_3_)	8.800 (7.500, 10.600)	8.740 (7.300, 10.620)	8.800 (7.740, 10.600)	Z = −0.588	0.557
CRP, M (Q_1_, Q_3_)	2.495 (0.910, 8.720)	1.900 (0.810, 5.810)	3.070 (1.075, 8.720)	Z = −3.084	0.002
UACR, M (Q_1_, Q_3_)	12.900 (5.450, 50.525)	6.000 (3.700, 12.050)	63.300 (37.200, 129.100)	Z = −13.576	<0.001
SATI, M (Q_1_, Q_3_)	42.855 (31.424, 62.324)	40.962 (29.916, 61.576)	45.192 (33.184, 62.873)	Z = −1.043	0.297
SATd, M (Q_1_, Q_3_)	−100.700 (−104.200, −96.807)	−100.600 (−104.450, −96.585)	−101.100 (−104.000, −97.450)	Z = −0.288	0.773
VATI, M (Q_1_, Q_3_)	53.539 (37.210, 70.147)	51.755 (35.661, 65.447)	56.020 (39.989, 75.322)	Z = −1.968	0.049
VATd, M (Q_1_, Q_3_)	−97.720 (−101.600, −92.527)	−97.400 (−101.650, −92.685)	−97.990 (−101.350, −92.565)	Z = −0.163	0.871
IMATI, M (Q_1_, Q_3_)	3.098 (2.036, 4.935)	2.995 (1.918, 4.262)	3.423 (2.235, 5.156)	Z = −2.278	0.023
IMATd, M (Q_1_, Q_3_)	−65.630 (−69.032, −61.998)	−64.930 (−68.715, −61.350)	−66.510 (−69.590, −63.015)	Z = −2.319	0.020
SMI, M (Q_1_, Q_3_)	47.759 (40.014, 55.144)	48.119 (40.527, 55.180)	47.704 (39.435, 54.663)	Z = −0.816	0.414
SMAd, M (Q_1_, Q_3_)	36.810 (31.312, 40.985)	37.670 (31.850, 41.305)	35.740 (30.150, 40.360)	Z = −2.153	0.031
RSFI, M (Q_1_, Q_3_)	0.954 (0.566, 1.494)	0.852 (0.456, 1.305)	1.206 (0.676, 1.799)	Z = −3.733	<0.001
RSFd, M (Q_1_, Q_3_)	−72.986 (−76.547, −69.592)	−71.381 (−74.255, −66.983)	−75.550 (−79.740, −72.085)	Z = −7.236	<0.001
PATI, M (Q_1_, Q_3_)	14.650 (8.894, 19.576)	13.515 (8.230, 18.507)	16.371 (10.781, 21.801)	Z = −3.174	0.002
PATd, M (Q_1_, Q_3_)	−93.540 (−97.988, −87.785)	−94.000 (−98.725, −88.385)	−93.160 (−96.920, −87.350)	Z = −1.854	0.064

**Note:**

t, t-test; Z, Mann-Whitney test; $\chi^2$, Chi-square test; SD, standard deviation; M, median; Q_1_, 1st quartile, Q_3_: 3st quartile. PATd, perirenal adipose tissue density; SATI, subcutaneous adipose tissue index; RSFI, renal sinus fat index; RSFd, renal sinus fat density; PATI, perirenal adipose tissue index; SATd, subcutaneous adipose tissue density; CRP, high-sensitivity C-reactive protein; VATI, visceral adipose tissue index; IMATI, intermuscular adipose tissue index; IMATd, intermuscular adipose tissue; SMAd, skeletal muscle area density; TC, total cholesterol; TG, triglycerides; LDL-C, low-density lipoprotein cholesterol; HDL-C, high-density lipoprotein cholesterol; CRP, C-reactive protein; HbA1C, glycated hemoglobin.

### Inter-observer reliability assessment

Intraclass correlation coefficients (ICC) with 95% confidence intervals for body composition parameters demonstrated excellent reproducibility: VATI (ICC = 0.967, 95% CI [0.932–0.984]); VATd (ICC = 0.931, 95% CI [0.862–0.967]); SATI (ICC = 1.000, 95% CI [1.000–1.000]); SATd (ICC = 0.995, 95% CI [0.990–0.998]); IMATI (ICC = 0.960, 95% CI [0.919–0.981]); IMATd (ICC = 0.885, 95% CI [0.775–0.944]); SMI (ICC = 0.949, 95% CI [0.897–0.975]); SMAd (ICC = 0.998, 95% CI [0.996–0.999]); RSFI (ICC = 0.968, 95% CI [0.936–0.985]); RSFd (ICC = 0.976, 95% CI [0.950–0.988]); PATI (ICC = 0.999, 95% CI [0.998–1.000]); PATd (ICC = 0.973, 95% CI [0.945–0.987]). All body composition parameters exhibited excellent inter-observer measurement reproducibility and reliability (ICC > 0.75). For details, see Table S2.

### Risk factor analysis for early DKD

Univariate logistic regression analysis identified significant associations with early DKD for: age (OR = 1.03, 95% CI [1.01–1.05], *P* = 0.001), hypertension history (OR = 2.78, 95% CI [1.75–4.44], *P* < 0.001), CRP (OR = 1.02, 95% CI [1.01–1.03], *P* = 0.014), IMATI (OR = 1.13, 95% CI [1.03–1.23], *P* = 0.008), IMATd (OR = 0.95, 95% CI [0.92–0.99], *P* = 0.025), SMAd (OR = 0.97, 95% CI [0.94–0.99], *P* = 0.036), RSFI (OR = 1.65, 95% CI [1.23–2.21], *P* < 0.001), RSFd (OR = 0.85, 95% CI [0.80–0.89], *P* < 0.001), and PATI (OR = 1.05, 95% CI [1.02–1.08], *P* = 0.001).

Multivariate logistic regression analysis using stepwise selection identified four independent risk factors for early DKD: age (OR = 1.03, 95% CI [1.01–1.06], *P* = 0.044), CRP (OR = 1.02, 95% CI [1.01–1.04], *P* = 0.005), RSFI (OR = 0.50, 95% CI [0.30–0.85], *P* = 0.010), and RSFd (OR = 0.79, 95% CI [0.74–0.85], *P* < 0.001) (Table 2).

**Table 2:** Logistic regression analysis of early DKD in T2DM patients.

Variables	Univariate analysis					Multivariate analysis				
	β	S.E	Z	*P*	OR (95% CI)	β	S.E	Z	*P*	OR (95% CI)
Age	0.03	0.01	3.26	0.001	1.03 [1.01–1.05]	0.03	0.02	2.01	0.044	1.03 [1.01–1.06]
Height	−1.14	1.42	−0.80	0.424	0.32 [0.02–5.20]					
Weight	0.01	0.01	0.77	0.440	1.01 [0.99–1.02]					
BMI	0.04	0.03	1.36	0.174	1.04 [0.98–1.10]					
Hypertension										
No					1.00 (Reference)					1.00 (Reference)
Yes	1.02	0.24	4.30	<0.001	2.78 [1.75–4.44]	0.58	0.31	1.90	0.058	1.79 [0.98–3.26]
Smoking										
No					1.00 (Reference)					
Yes	0.09	0.26	0.35	0.725	1.10 [0.66–1.82]					
Alcohol consumption										
No					1.00 (Reference)					
Yes	0.25	0.30	0.83	0.407	1.28 [0.71–2.30]					
Gender										
Female					1.00 (Reference)					
Male	0.01	0.24	0.03	0.975	1.01 [0.63–1.60]					
Systolic pressure	0.01	0.01	1.48	0.140	1.01 [1.00–1.02]					
Diastolic pressure	0.00	0.01	0.38	0.706	1.00 [0.98–1.02]					
TG	0.07	0.08	0.89	0.372	1.08 [0.92–1.26]					
TC	−0.07	0.10	−0.75	0.451	0.93 [0.76–1.13]					
LDL-C	−0.14	0.13	−1.02	0.306	0.87 [0.67–1.13]					
HDL-C	−0.48	0.40	−1.19	0.234	0.62 [0.28–1.36]					
Urea	0.07	0.05	1.36	0.173	1.08 [0.97–1.19]					
Uric acid	0.00	0.00	0.47	0.636	1.00 [1.00–1.00]					
Serum C-peptide	0.02	0.07	0.27	0.789	1.02 [0.89–1.17]					
HbA1c	0.02	0.05	0.45	0.654	1.02 [0.92–1.13]					
CRP	0.02	0.01	2.46	0.014	1.02 [1.01–1.03]	0.02	0.01	2.81	0.005	1.02 [1.01–1.04]
SATI	0.01	0.00	1.39	0.165	1.01 [1.00–1.01]					
SATd	−0.00	0.01	−0.12	0.905	1.00 [0.97–1.03]					
VATI	0.01	0.00	1.77	0.077	1.01 [1.00–1.02]					
VATd	0.01	0.02	0.41	0.684	1.01 [0.97–1.04]					
IMATI	0.12	0.05	2.64	0.008	1.13 [1.03–1.23]	−0.02	0.09	−0.25	0.805	0.98 [0.81–1.18]
IMATd	−0.05	0.02	−2.25	0.025	0.95 [0.92–0.99]	−0.01	0.03	−0.19	0.847	0.99 [0.93–1.06]
SMI	−0.01	0.01	−0.93	0.351	0.99 [0.97–1.01]					
SMAd	−0.03	0.02	−2.10	0.036	0.97 [0.94–0.99]	−0.00	0.04	−0.09	0.926	1.00 [0.93–1.07]
RSFI	0.50	0.15	3.33	<0.001	1.65 [1.23–2.21]	−0.69	0.27	−2.57	0.010	0.50 [0.30–0.85]
RSFd	−0.17	0.03	−6.48	<0.001	0.85 [0.80–0.89]	−0.23	0.04	−6.31	<0.001	0.79 [0.74–0.85]
PATI	0.05	0.01	3.27	0.001	1.05 [1.02–1.08]	0.01	0.02	0.35	0.724	1.01 [0.96–1.06]
PATd	0.02	0.01	1.93	0.053	1.02 [1.00–1.05]					

**Note:**

OR, odds ratio; CI, confidence interval. PATd, perirenal adipose tissue density; SATI, subcutaneous adipose tissue index; RSFI, renal sinus fat index; RSFd, renal sinus fat density; PATI, perirenal adipose tissue index; SATd, subcutaneous adipose tissue density; CRP, high-sensitivity C-reactive protein; VATI, visceral adipose tissue index; IMATI, intermuscular adipose tissue index; IMATd, intermuscular adipose tissue; SMAd, skeletal muscle area density; TC, total cholesterol; TG, triglycerides; LDL-C, low-density lipoprotein cholesterol; HDL-C, high-density lipoprotein cholesterol; CRP, C-reactive protein; HbA1C, glycated hemoglobin.

### Subgroup analysis

**Figure 3:**
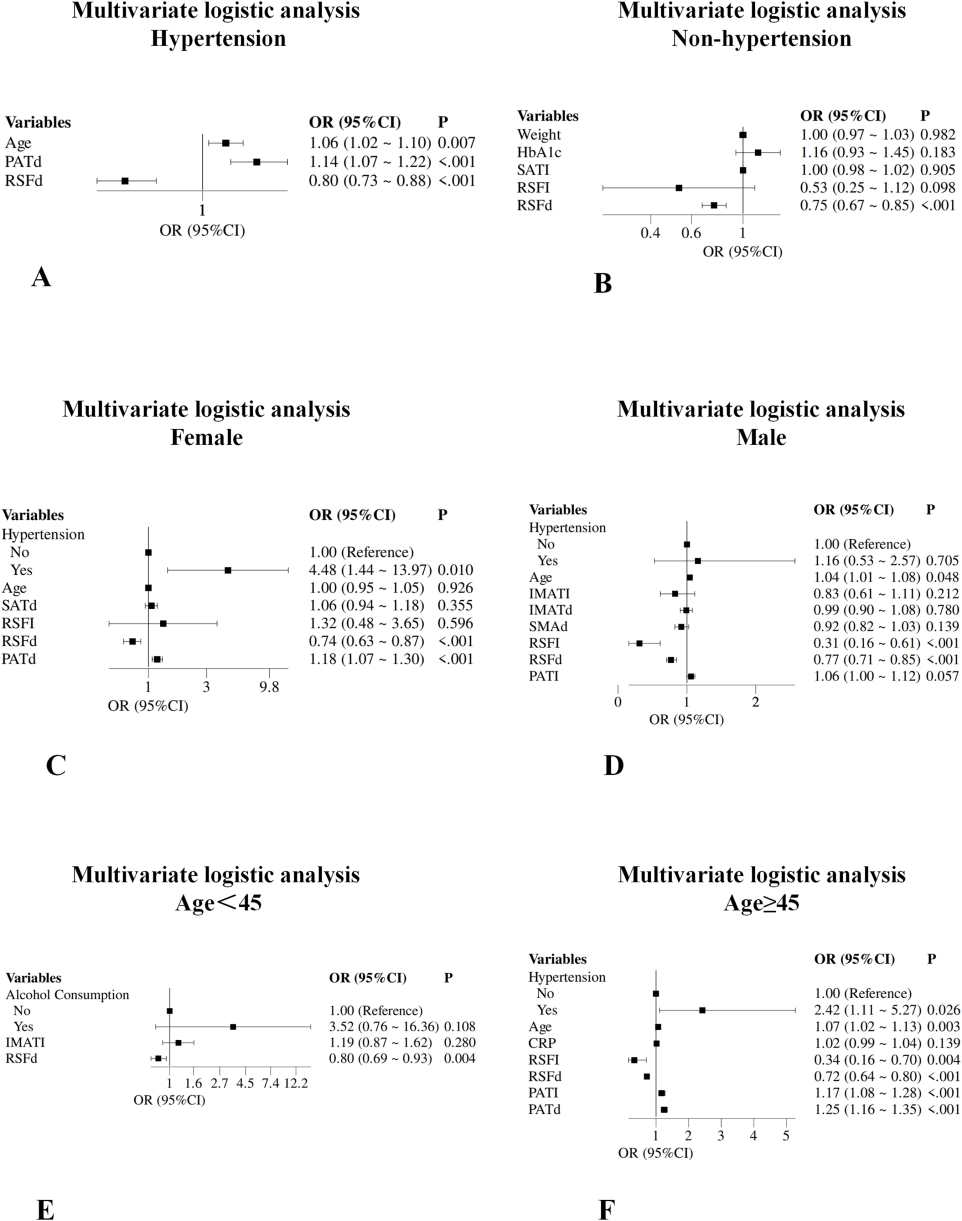
Subgroup analysis forest plot. The forest plot shows the predictive ability of independent factors obtained by multivariate survival analysis in hypertensive (A), non-hypertensive (B), female (C), male (D), age < 45 (E), and age ≥ 45 (F) subgroups. PATd, perirenal adipose tissue density; BMI, body mass index; SATI, subcutaneous adipose tissue index; RSFI, renal sinus fat index; RSFd, renal sinus fat density; PATI, perirenal adipose tissue index; SATd, subcutaneous adipose tissue density; CRP, high-sensitivity C-reactive protein; VATI, visceral adipose tissue index; IMATI, intermuscular adipose tissue index; IMATd, intermuscular adipose tissue; SMAd, skeletal muscle area density.

Multivariate logistic regression subgroup analysis showed that RSFd had a consistent protective effect across all subgroups. Additionally, in male patients, age and PATI (OR = 1.06, *P* = 0.057) were independent risk factors; in females, hypertension (OR = 4.48, *P* = 0.010) and PATd (OR = 1.18, *P* < 0.001) were independent risk factors. In the hypertension stratification, the non-hypertensive group had only RSFd as a protective factor; in the hypertensive group, besides RSFd, age (OR = 1.06, *P* = 0.007) and PATd (OR = 1.14, *P* < 0.001) were risk factors. Age stratification showed that in patients aged ≥45 years, besides RSFd (OR = 0.72, *P* < 0.001), hypertension, age, PATI, and PATd were all risk factors (Fig. 3).

### Correlations between body composition parameters and renal function

Spearman correlation analysis showed that RSTd was weakly negatively correlated with UACR (r = −0.31, *P* < 0.001), while RSFI was weakly positively correlated with UACR (r = 0.20, *P* < 0.001). Additionally, SMAd was weakly negatively correlated with UACR (r = −0.17, *P* = 0.002), and PATI was weakly positively correlated with UACR (r = 0.18, *P* = 0.001). Regarding eGFR, MATI showed a weak negative correlation (r = −0.13, *P* = 0.023), while MATd showed a marginal negative correlation (r = −0.10, *P* = 0.067). VATI was weakly positively correlated with UACR (r = 0.12, *P* = 0.035). No other adipose or muscle composition indicators showed statistically significant correlations with UACR or eGFR in this dataset (Fig. S1).

Multiple linear regression analysis after adjusting for confounding variables demonstrated that RSFd was the only parameter maintaining significant associations with both renal function indicators: negative correlation with UACR (β = −1.88, *P* < 0.001) and positive correlation with eGFR (β = 0.22, *P* = 0.017) (Table 3).

**Table 3:** Multiple linear regression analysis for renal function indicators.

Renal function indicators	Variables	Model 1		Model 2		Model 3	
		β (95% CI)	*P*	β (95% CI)	*P*	β (95% CI)	*P*
UACR, mg/g	VATI, cm^2^/m^2^	0.13 [−0.13 to 0.39]	0.333	−0.18 [−0.58 to 0.22]	0.373	0.21 [−0.28 to 0.71]	0.396
	IMATI, cm^2^/m^2^	2.41 [−0.19 to 5.00]	0.070	0.11 [−3.09 to 3.31]	0.945	−1.58 [−5.31 to 2.15]	0.408
	SMAd, HU	−1.32 [−2.25 to −0.39]	0.006	−0.42 [−1.80 to 0.97]	0.557	−0.59 [−2.17 to 0.98]	0.459
	RSFI, cm^2^/m^2^	6.77 [−1.78 to 15.31]	0.122	1.27 [−8.63 to 11.16]	0.802	7.16 [−3.19 to 17.51]	0.176
	RSFd, HU	−1.67 [−2.44 to −0.89]	<0.001	−1.35 [−2.20 to −0.51]	0.002	−1.88 [−2.76 to −1.00]	<0.001
	PATI, cm^2^/m^2^	0.81 [−0.03 to 1.64]	0.059	0.20 [−0.89 to 1.30]	0.716	2.08 [0.71 to 3.45]	0.003
eGFR, ml/min/1.73 m^2^	VATI, cm^2^/m^2^	−0.06 [−0.11 to −0.01]	0.024	−0.05 [−0.13 to 0.03]	0.194	−0.08 [−0.18 to 0.03]	0.148
	SMI, cm^2^/m^2^	−0.17 [−0.30 to −0.04]	0.012	−0.02 [−0.27 to 0.23]	0.894	−0.03 [−0.28 to 0.23]	0.838
	SMAd, HU	−0.12 [−0.29 to 0.06]	0.199	−0.01 [−0.28 to 0.26]	0.931	0.05 [−0.34 to 0.44]	0.816
	RSFI, cm^2^/m^2^	−1.46 [−3.06 to 0.14]	0.075	−1.03 [−2.96 to 0.89]	0.294	−1.27 [−3.38 to 0.85]	0.241
	RSFd, HU	0.26 [0.11 to 0.41]	<0.001	0.24 [0.08 to 0.41]	0.004	0.22 [0.04 to 0.40]	0.017
	PATI, cm^2^/m^2^	−0.17 [−0.32 to −0.01]	0.035	−0.09 [−0.31 to 0.12]	0.396	−0.18 [−0.46 to 0.10]	0.199

**Note:**

CI: Confidence Interval Model 1: without adjustment. Model 2: a. Adjustment for hypertension, smoking, alcohol consumption, gender, age, height, weight, BMI, systolic pressure, diastolic pressure, TG, TC, LDL-C, HDL-C, eGFR, urea, uric acid, serum C-peptide, HbA1c, and CRP. B. adjustment for hypertension, smoking, alcohol consumption, gender, age, height, weight, BMI, systolic pressure, diastolic pressure, TG, TC, LDL-C, HDL-C, eGFR, urea, uric acid, serum C-peptide, HbA1c, and CRP. Model 3: a. Adjustment for SATI, SATd, VATI, VATd, IMATd, SMI, and PATd in addition to the variables in model 2a. b. Adjustment for SATI, SATd, IMATI, IMATd, SMd, PATId, and RSFI in addition to the variables in model 2b.

### Predictive model development and validation

**Figure 4:**
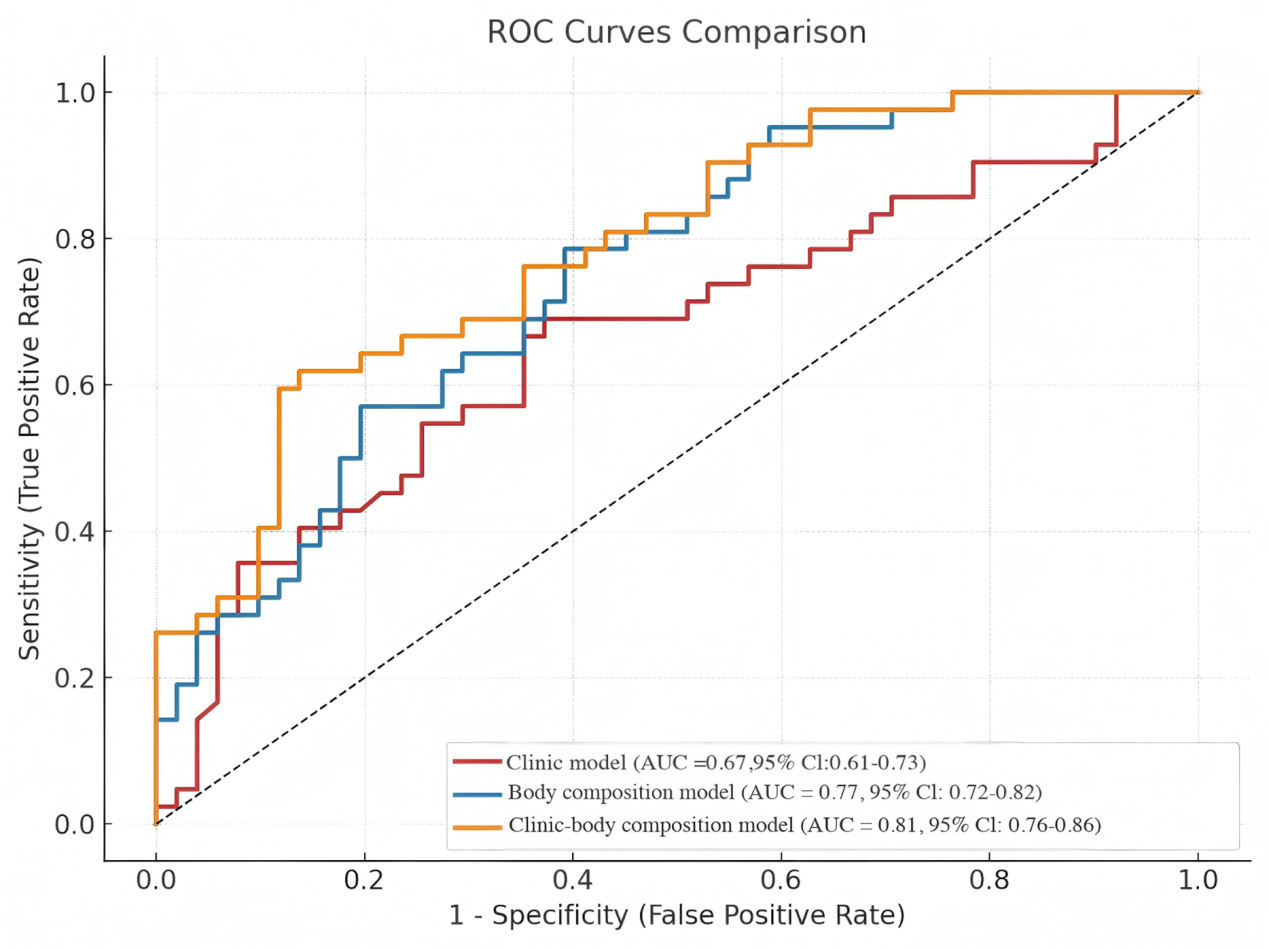
ROC curves for predicting early DKD in T2DM patients using clinical parameters and body composition parameters. ROC, receiver operating characteristic; AUC, the area under ROC curve.

Based on multivariate logistic regression results, three predictive models were constructed: body composition parameter model, clinical parameter model, and combined clinical-body composition model. ROC curve analysis demonstrated: Clinical model AUC: 0.67 (95% CI [0.61–0.73]); Body composition parameter model AUC: 0.77 (95% CI [0.72–0.82]); Combined model AUC: 0.81 (95% CI [0.76–0.86]). The combined model achieved significantly superior predictive performance compared to individual models, indicating that body composition parameters substantially enhance early DKD risk prediction capability (See Fig. 4 and Table 4).

**Table 4:** Predictive value of clinical parameters and body composition parameters for early DKD in T2DM patients.

	AUC (95% CI)	Accuracy (95% CI)	Sensitivity (95% CI)	Specificity (95% CI)	PPV (95% CI)	NPV (95% CI)	Cut off
Clinic model	0.67 [0.61–0.73]	0.68 [0.62–0.73]	0.78 [0.72–0.84]	0.53 [0.45–0.62]	0.70 [0.63–0.76]	0.64 [0.55–0.73]	0.500
Body composition model	0.77 [0.72–0.82]	0.71 [0.66–0.76]	0.74 [0.68–0.81]	0.66 [0.58–0.74]	0.75 [0.69–0.82]	0.65 [0.57–0.73]	0.454
Clinical–body composition model	0.81 [0.76–0.86]	0.74 [0.68–0.78]	0.74 [0.67–0.80]	0.73 [0.66–0.81]	0.79 [0.73–0.85]	0.67 [0.59–0.75]	0.423

**Note:**

The cut-off values in these analyses were determined by maximizing the Youden index, a standard method for identifying optimal thresholds in diagnostic tests. ROC, receiver operating characteristic; AUC, the area under the ROC curve; CI, confidence interval.

The AUC was compared among three models using DeLong’s test. The results demonstrated that the body composition parameter model and the combined clinical-body composition model exhibited comparable predictive performance (Table S1).

## Discussion

This systematic abdominal CT body composition analysis represents the first study to identify RSFd as being independently associated with early DKD in patients with T2DM. Our findings demonstrate that lower RSFd is significantly associated with early DKD (OR = 0.79, *P* < 0.001). This association remained consistent across subgroups stratified by gender, age, and hypertension status, supporting its potential utility as an imaging biomarker.

RSF, situated within the renal sinus and fibrous membrane (Liu, Sun & Kong, 2019; Irazabal & Eirin, 2016), may link obesity to DKD by compressing sinus structures, potentially raising blood pressure and impairing renal function. This view is supported by Spit et al. (2020) who first linked higher RSF volume in T2DM patients to reduced eGFR and increased renal vascular resistance, suggesting a role in hypertension. These findings are consistent with our study, wherein increased renal sinus fat was an independent risk factor for early DKD.

The radiodensity of adipose tissue measured by CT is a reliable indicator of its biological function. White adipose tissue, as the primary energy storage organ, exhibits abnormal intracellular lipid accumulation when its function is impaired. This leads to an increased proportion of lipids per unit volume, while high-density components such as water and proteins relatively decrease, ultimately resulting in lower CT values (Wang et al., 2023; Proença et al., 2014). Therefore, the reduced renal sinus fat density observed in early DKD patients in this study likely reflects the pathological state of lipid accumulation and functional disorder. In addition, lipid overload can also lead to changes in renal sinus fat density through a series of complex pathophysiological pathways, including oxidative stress, mitochondrial dysfunction, inflammation, and fibrosis (Santillana et al., 2023).

Notably, our findings contrast with Liao et al. (2024), who reported that elevated RSFd increased T2DM renal dysfunction risk. These differences may stem from several methodological variations: in terms of the population, this study was limited to early DKD patients, whereas Liao et al.’s (2024) study included participants with a broader range of renal function impairment; regarding thresholds, this study defined RSF in the range of −150 to −50 HU, while Liao et al. (2024) used a wider range of −190 to −30 HU, which could affect density values; the study endpoints also fundamentally differed, as this study was cross-sectional, aiming to explore the association between RSFd and early DKD, whereas Liao et al. (2024) conducted a longitudinal analysis to assess the predictive value of RSFd for the risk of chronic kidney disease progression. Additionally, Fan et al. (2024) identified associations between reduced skeletal muscle density and early DKD but did not incorporate RSF parameters, resulting in different conclusions.

Our investigation revealed that conventional BMI was not an independent risk factor for early DKD in T2DM patients (*P* > 0.05), contrasting with Garofalo et al. (2017). This discrepancy may be attributed to BMI limitations as a global obesity indicator: inability to distinguish muscle from adipose tissue or quantify specific fat distribution patterns and quality characteristics (Li et al., 2022). This finding emphasizes that early DKD development may relate to the heterogeneous distribution of specific adipose depots rather than overall obesity status.

Regarding abdominal fat distribution and renal function relationships, our study confirmed mild correlations between VAT and PAT with renal function indicators. However, neither emerged as an independent risk factor for early DKD. VAT induces insulin resistance, mitochondrial dysfunction, and oxidative stress through abundant adipokine production, ultimately causing renal injury (Ziegler & Scheele, 2024; Hall et al., 2021). PAT, representing specialized VAT surrounding kidneys, may demonstrate closer DKD associations due to unique anatomical positioning and developmental heterogeneity (Zbrzeźniak-Suszczewicz et al., 2025). However, our results suggest VAT and PAT may indirectly influence renal function through interactions with multiple metabolic factors rather than directly causing early DKD.

We observed weak negative correlations between SMI and eGFR, and between SMAd and UACR, suggesting associations between muscle loss, muscle fat infiltration, and renal dysfunction, consistent with previous research (Avesani et al., 2023; Huang et al., 2022). However, these correlations were relatively weak, possibly related to our study population comprising early DKD patients with relatively slight renal function indicator variations, some within normal ranges.

Our established multifactor predictive model incorporated four independent risk factors: age, CRP, RSFI, and RSFd. Notably, RSFI exhibited opposite association directions in univariate *vs* multivariate analyses: univariate analysis showed elevated RSFI increased DKD risk (OR = 1.65), while multivariate analysis yielded OR = 0.50, suggesting RSFI effects on early DKD risk are modulated by other confounding factors (Kaneko et al., 2024).

CRP, as an inflammatory marker, may exacerbate renal tissue injury and fibrosis through transforming growth factor-β (TGF-β)/Smad3 signaling pathways (Tang et al., 2022). Age, as a non-modifiable risk factor, demonstrates well-established associations with DKD development (Fabre & Rangel, 2024). These findings further support the critical roles of inflammation and aging in early DKD pathogenesis.

Subgroup analyses demonstrated RSFd as an independent risk factor across all subgroups, emphasizing stable predictive value. Additionally, in hypertensive, age ≥ 45 and female patient subgroups, PATd emerged as an independent risk factor, suggesting that in patients with hypertension, age ≥ 45, and in female patients, special attention should be paid to the elevation of PATd, potentially related to adipose tissue-associated inflammatory mechanisms (Kashiwagi, 2025).

Through ROC curve analysis, we observed that the model based only on clinical parameters produced an AUC of 0.67 (95% CI [0.61–0.73]). In contrast, the body composition parameter model exhibited meaningfully higher discriminative performance, with an AUC of 0.77 (95% CI [0.72–0.82]). Most importantly, the combined clinical–body composition model achieved the highest predictive accuracy, with an AUC of 0.81 (95% CI [0.76–0.86]). These findings underscore the value of incorporating body composition metrics into conventional clinical models to improve early detection of DKD in T2DM patients.

Several limitations warrant acknowledgment: First, although we ensured that the selected patients did not have COVID-19 in their discharge diagnoses, all study subjects were recruited during the COVID-19 pandemic, a period of universal susceptibility. Coupled with evidence of poorer outcomes in chronic kidney disease patients with COVID-19, this contextual factor may potentially influence the study results (Chen et al., 2020; Yang et al., 2020). Second, this single-center retrospective study has a limited sample size, and its models lack internal validation; these factors may collectively compromise the generalizability of the findings. Therefore, future multicenter, large-scale prospective studies or external validation are required to further confirm the findings of this research. Third, the regression models developed in this study were not subjected to internal validation, which may lead to over-optimistic estimates of their performance; therefore, external validation is required to confirm their generalizability. Fourth, while CT represents the gold standard for body composition assessment, the inherent ionizing radiation exposure limits its routine application in healthy populations. However, opportunistic body composition evaluation in patients receiving abdominal CT for other medical indications provides significant clinical value. Finally, inclusion criteria based on renal dysfunction may miss occult DKD patients with negative laboratory findings; future research could incorporate renal biopsy pathological diagnosis for enhanced study design.

## Conclusions

Through systematic abdominal CT body composition analysis, this study found that renal sinus fat density is an independent factor associated with the risk of early diabetic nephropathy in patients with type 2 diabetes. The multifactorial model, which includes RSFd, RSFI, age, and CRP, performed well and may provide potential imaging indicators for the identification and risk assessment of early diabetic nephropathy. Despite certain limitations, this study offers new insights for the screening of early diabetic nephropathy. Future multicenter prospective studies are necessary to validate the clinical applicability of these findings and further explore the role of body composition parameters in the diagnosis and intervention strategies of early diabetic nephropathy.

## Supplemental Information

10.7717/peerj.20535/supp-1 Supplemental Information 1 Univariate correlation analysis of eGFR and UACR with body composition parameters. (A) Correlation analysis between eGFR and body composition. (B) Correlation analysis between UACR and body composition.

10.7717/peerj.20535/supp-2 Supplemental Information 2 Comparing the AUC under different ROC curves using DeLong’s test. The differences in predictive performance (AUC) between three different models (Clinic, Body composition, and Combined), along with their 95% confidence intervals and corresponding *P*-values.

10.7717/peerj.20535/supp-3 Supplemental Information 3 Intraclass correlation coefficients. ICC: Intraclass correlation coefficients; All body composition parameters exhibited excellent inter-observer measurement reproducibility and reliability (ICC >0.75).

10.7717/peerj.20535/supp-4 Supplemental Information 4 Original Dataset. The raw anonymized data underlying all analyses of this study, including all subjects’ basic clinical indicators, body composition parameters, and renal function assessment results.

10.7717/peerj.20535/supp-5 Supplemental Information 5 Interpret numerical values. An explanation of the numbers in the categorical variables.

10.7717/peerj.20535/supp-6 Supplemental Information 6 STROBE Documentation.

## Additional Information and Declarations

### Data Availability

The following information was supplied regarding data availability:

The raw data is available in the Supplemental Files.
