# Supplementary figures and images for: Association between abdominal CT-based body composition parameters and early diabetic kidney disease in type 2 diabetes mellitus: a retrospective cross-sectional study
Source: PeerJ. 2026 Jan 15;14:e20535. doi: 10.7717/peerj.20535 (PMC12812273; doi:10.7717/peerj.20535)

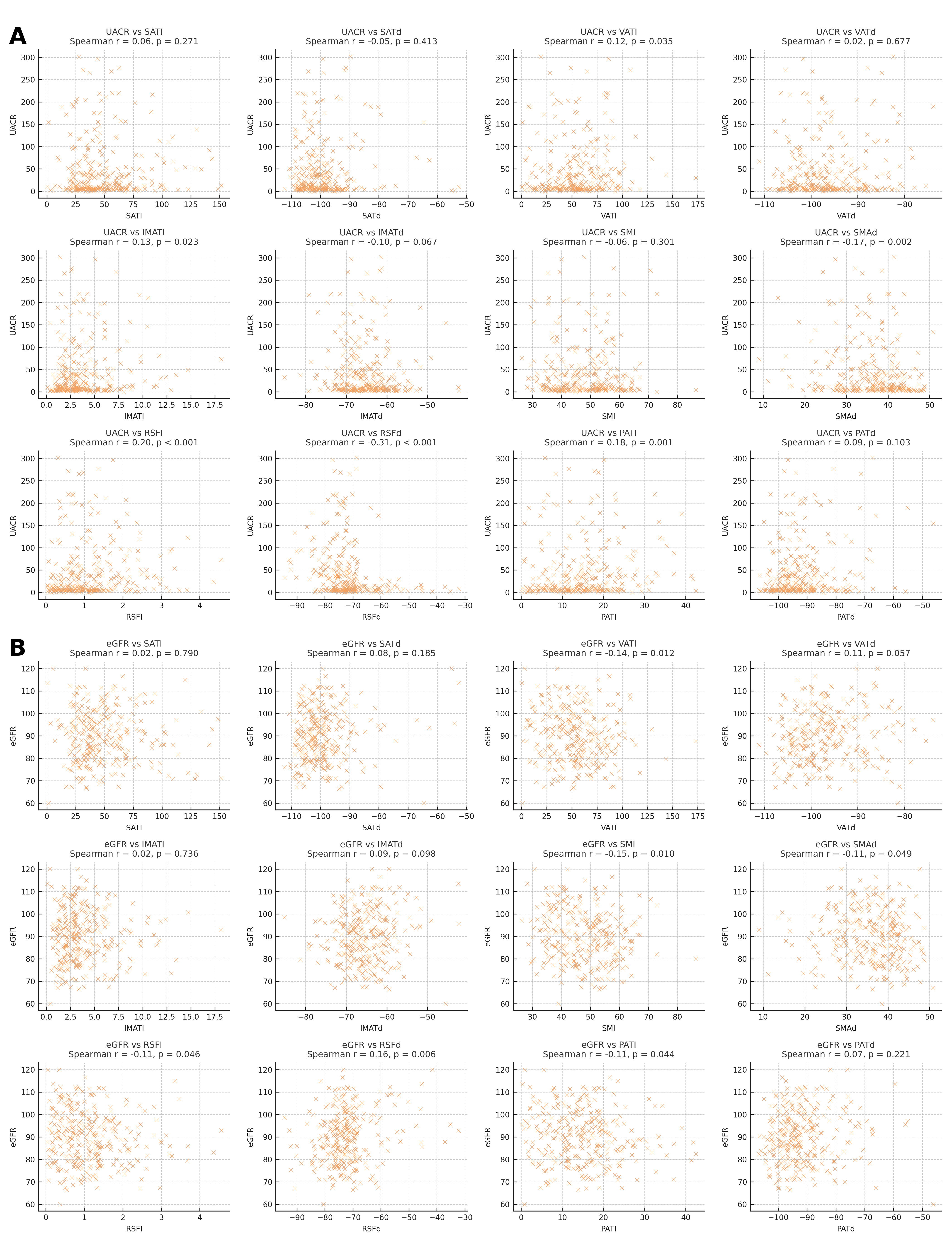

Supplement: Supplemental Information 1 — (A) Correlation analysis between eGFR and body composition. (B) Correlation analysis between UACR and body composition. [file peerj-14-20535-s001.png]
