# Supplementary material for: Association between abdominal CT-based body composition parameters and early diabetic kidney disease in type 2 diabetes mellitus: a retrospective cross-sectional study
Source: PeerJ. 2026 Jan 15;14:e20535. doi: 10.7717/peerj.20535 (PMC12812273; doi:10.7717/peerj.20535)
Supplement: Supplemental Information 2 — The differences in predictive performance (AUC) between three different models (Clinic, Body composition, and Combined), along with their 95% confidence intervals and corresponding P-values. [file peerj-14-20535-s002.docx]

|  | **Difference between AUC (95% CI)** | | **p-value** |
| --- | --- | --- | --- |
| **Clinic vs Body composition** | | 0.085 (-0.007, 0.176) | 0.272 |
| **Clinic vs Combined** | | 0.123 (0.034, 0.213) | 0.099 |
| **Body composition vs Combined** | | 0.039 (-0.047, 0.124) | 0.584 |
|  | | | |
