# Supplementary material for: Association between abdominal CT-based body composition parameters and early diabetic kidney disease in type 2 diabetes mellitus: a retrospective cross-sectional study
Source: PeerJ. 2026 Jan 15;14:e20535. doi: 10.7717/peerj.20535 (PMC12812273; doi:10.7717/peerj.20535)
Supplement: Supplemental Information 3 — ICC: Intraclass correlation coefficients; All body composition parameters exhibited excellent inter-observer measurement reproducibility and reliability (ICC >0.75). [file peerj-14-20535-s003.docx]

| **Variables** | | **ICC** | **95%CI** | **Explanation** | |
| --- | --- | --- | --- | --- | --- |
| VATI  VATd  SATI  SATd  IMATI  IMATd  SMI  SMAd  RSFI  RSFd  PATI  PATd | | 0.967  0.931  1.000  0.995  0.960  0.885  0.949  0.998  0.968  0.976  0.999  0.973 | 0.932-0.984  0.862-0.967  1.000-1.000  0.990-0.998  0.919-0.981  0.775-0.944  0.897-0.975  0.996-0.999  0.936-0.985  0.950-0.988  0.998-1.000  0.945-0.987 | Excellent consistency  Excellent consistency  Excellent consistency  Excellent consistency  Excellent consistency  Good consistency  Excellent consistency  Excellent consistency  Excellent consistency  Excellent consistency  Excellent consistency  Excellent consistency | |
|  |  | | | |  |
