# Supplementary material for: Association between abdominal CT-based body composition parameters and early diabetic kidney disease in type 2 diabetes mellitus: a retrospective cross-sectional study
Source: PeerJ. 2026 Jan 15;14:e20535. doi: 10.7717/peerj.20535 (PMC12812273; doi:10.7717/peerj.20535)
Supplement: Supplemental Information 5 — An explanation of the numbers in the categorical variables. [file peerj-14-20535-s005.docx]

In the context of categorical variables, the term "label" refers to the grouping used in the study. The label is used to distinguish between two groups within the study population:

**Label = 1:** This indicates the group of patients with Type 2 Diabetes Mellitus (T2DM) who also have early Diabetic Kidney Disease (DKD).

**Label = 0:** This indicates the group of patients with Type 2 Diabetes Mellitus (T2DM) who do not have Diabetic Kidney Disease (DKD).
